# Role of the IL-23/IL-17 Axis in Psoriasis and Psoriatic Arthritis: The Clinical Importance of Its Divergence in Skin and Joints

**DOI:** 10.3390/ijms19020530

**Published:** 2018-02-09

**Authors:** Marie-Astrid Boutet, Alessandra Nerviani, Gabriele Gallo Afflitto, Costantino Pitzalis

**Affiliations:** 1 Centre for Experimental Medicine & Rheumatology, William Harvey Research Institute and Barts and The London School of Medicine and Dentistry, Queen Mary University of London, London EC1M 6BQ, UK; m.a.boutet@qmul.ac.uk (M.-A.B.); a.nerviani@qmul.ac.uk (A.N.); 2 Unit of Allergology, Immunology & Rheumatology, Department of Medicine, Università campus Bio-Medico di Roma, 00128 Rome, Italy; gabgallo9@gmail.com

**Keywords:** psoriasis, psoriatic arthritis, interleukin-17, interleukine-23, Th17 cells

## Abstract

Psoriasis is a chronic systemic inflammatory disease causing erythematosus and scaly skin plaques; up to 30% of patients with psoriasis develop Psoriatic Arthritis (PsA), which is characterised by inflammation and progressive damage of the peripheral joints and/or the spine and/or the entheses. The pathogenic mechanisms driving the skin disorder in psoriasis and the joint disease in PsA are sustained by the activation of inflammatory pathways that can be overlapping, but also, at least partially, distinct. Cytokines members of the IL-23/IL-17 family, critical in the development of autoimmunity, are abundantly expressed within the cutaneous lesions but also seem to be involved in chronic inflammation and damage of the synovium though, as it will be here discussed, not in all patients. In this review, we will focus on the state of the art of the molecular features of psoriatic skin and joints, focusing on the specific role of the IL-23/IL-17 pathway in each of these anatomical districts. We will then offer an overview of the approved and in-development biologics targeting this axis, emphasising how the availability of the “target” in the diseased tissues could provide a plausible explanation for the heterogeneous clinical efficacy of these drugs, thus opening future perspective of personalised therapies.

## 1. Introduction

Psoriasis (Ps) is a systemic disease that affects around 1% of the population worldwide [1] causing chronic inflammation of the skin. As described below, although several Ps clinical subtypes have been described (guttate, pustular, and erythrodermic), classical plaque psoriasis is characterised by the focal formation of inflamed, red and raised plaques secondary to excessive growth of skin epithelial cells. In up to 30% of patients with skin psoriasis, a chronic seronegative spondyloarthropathy defined Psoriatic Arthritis (PsA) may develop; this is characterised by the presence of spondylitis, enthesitis or peripheral arthritis [2]. Despite being once considered as a relatively benign disease, it is now recognised that PsA is a systemic disorder with potentially severe manifestations beyond joint and skin involvement such as cardiovascular comorbidities and increased risk of cancer [3,4]. The diagnosis of Ps is primarily clinical, and it can be made by taking the patient’s history and through a meticulous evaluation of the skin, nails and scalp. Several clinical phenotypes have been described depending on the morphology of the cutaneous lesions and their anatomical locations: among all, plaque psoriasis is the most commonly found, affecting 80–90% of all psoriatic patients [5,6]. In the absence of specific diagnostic criteria, the diagnosis of PsA, particularly in the context of clinical trials, relies on the classification criteria published by the CASPAR (ClASsification criteria for Psoriatic ARthritis) group, which include: (i) evidence of psoriasis; (ii) psoriatic nail dystrophy; (iii) negative tests for rheumatoid arthritis (RA); (iv) dactylitis; and (v) radiographic evidence of juxta-articular new bone formation [7]. Patients meeting three of the five criteria are diagnosed with PsA. Overall, the CASPAR criteria have a specificity of 98.7% and a sensitivity of 91.4%. Among the various clinical subtypes of Ps, none has been specifically associated with the occurrence of PsA; nonetheless, the presence of nail dystrophy and intergluteal/perianal lesions, as well as the severity of the skin involvement, confer a significantly higher risk of developing PsA [8]. Several clinical patterns of PsA have been described [9]. The peripheral joint disease can occasionally be reminiscent of the rheumatoid phenotype (polyarticular and symmetrical), however an asymmetrical mono/oligoarthritis is more commonly observed. The prevalent involvement of the distal interphalangeal joints is quite typical and helps to differentiate peripheral PsA from RA. Less frequently, PsA occurs as a more severe destructive and deforming disease known as PsA *mutilans* [10]. Overall, around 50% of patients affected by PsA may show axial manifestations such as spondylitis and sacroiliitis [11]. Moreover, inflammation of the entheses (enthesitis) and dactylitis are frequently found in PsA patients [12]. The inclusion of the biologic agents into the strategy for the management of Ps and PsA has undoubtedly improved the disease’s outcome. Nevertheless, a considerable proportion of patients, especially those suffering from articular manifestations, do not adequately respond to treatment, therefore highlighting the impelling need to enhance the understanding of the pathophysiology and to define prognostic and predictive markers of disease evolution and treatment response, eventually paving the way towards a “personalised” therapeutic approach.

The pro-inflammatory cytokine IL-23, composed by the two subunits p19 and p40, is mainly produced by inflammatory Dendritic Cells (DCs) within the inflamed skin [13], with the additional contribution of macrophages and keratinocytes [14,15]. IL-23 induces the expansion and the maintenance of the T helper (Th) 17 subsets of T cells. Th17 lymphocytes are characterised by the expression of the transcription factor Retinoic acid receptor-Related Orphan receptor-γt (ROR-γt), typically produce the cytokine IL-17, and display a considerable degree of context-dependent plasticity. Targeting the IL-23/IL-17 axis has been shown to be a winning strategy in both Ps and PsA, as demonstrated by the clinical efficacy of the antagonists currently in use and by the ongoing development of new agents.

It is important, however, to note the discordant effectiveness between skin and joint disease at least in a sizable number of patients. Here, therefore, we will provide an update of the recent advances in the understanding of Ps/PsA pathophysiology, including the tissue-dependent selective role of the IL-23/IL-17 axis, and the latest knowledge about approved and in-trial therapeutics targeting this pathway.

## 2. Psoriasis and Psoriatic Arthritis, Same Disease?

Both Ps and PsA are chronic multifactorial diseases driven by a complex interplay between genetic factors, environment and immune dysfunction. In this section, we will review and highlight their similarities and differences with regards to pathogenesis, metabolic biomarkers and histological features.

### 2.1. Common and Disease-Specific Genetic Factors

Ps and PsA share a partially overlapping genetic susceptibility, as suggested by the significant percentage (around 30%) of patients affected by skin psoriasis who develop PsA. Interestingly, even patients with the sole first-degree familiarity for Ps but no history of personal skin disease may exhibit clinical features of PsA [16]. Furthermore, monozygotic twins show a concordance rate for Ps ranging from 20% to 64% according to the different reports; overall, genetic factors seem to account for around 70% of the variation in the susceptibility to Ps [17]. A solid body of evidence has documented the implication in the pathogenesis of Ps of both the Human Leukocyte Antigen (HLA)-associated and non-HLA genes. Among the latter, genes regulating the epithelial differentiation within the skin, genes associated with the Th17 and the Tumour Necrosis Factor (TNF) signalling pathways, as well as genes controlling the Nuclear Factor-Kappa B (NF-κB) activation have been all related to the occurrence of psoriatic manifestations [18]. Conversely, a rare genetic variant of the Interferon-Induced with Helicase C Domain 1 (*IFIH1*) gene, encoding a protein implicated in the host response to viruses and fungi [19], seems to be protective not only for type 1 diabetes and Ps, as demonstrated a few years ago [20], but also for PsA [21]. Even if Ps and PsA share several genetic risk loci, disease-specific mutations have also been researched in the attempt to better understand the differences between the skin and the joint involvement. For instance, among the HLA-genes, some alleles of the *HLA-B* (B*08, B*27, B*38, and Bw4) have been found to be exclusively associated with PsA; moreover, a specific PsA-linked variant distinct from the well-known Ps-related susceptibility locus has been identified within the *IL-23R* gene. Another potential PsA-associated candidate risk gene (*SLC22A5*) has been recently described at the chromosome 5q31 [22,23]. Furthermore, Genome-Wide Association Studies (GWAS) revealed a significant association between the gene *PTPN22* and the risk of developing PsA, but not skin psoriasis [24]. On the other hand, the HLA-C*06 is strongly associated with Ps and predicts better clinical response to methotrexate (MTX) [25] and the IL-12/IL-23 antagonist ustekinumab [26] in psoriatic patients. Early data suggested a link also with PsA [27], however, Bowes and colleagues [28] lately confirmed the association with Ps but not with PsA. Conversely, HLA-B*27 has been recognised to be the most important risk factor for the spondylitic form of PsA [28], whereas the DR4 haplotype is more associated with the rheumatoid-like pattern of PsA [29].

### 2.2. The Role of Soluble Biomarkers in Psoriasis and Psoriatic Arthritis

Some peripheral blood biomarkers have been reported as useful in evaluating the disease severity and the response to treatment and for identifying psoriatic patients who are more likely to develop Ps-related arthritis [30]. For instance, an increase in urea cycle amino acids (e.g., arginine and citrulline) and in amino acids required for the synthesis of collagen (e.g., hydroxyproline) correlates with more severe manifestations of Ps, usually assessed by the Psoriasis Area and Severity Index (PASI) score. Consistently, circulating levels of these metabolites tend to normalise in response to successful treatment [31]. Patients affected by PsA generally show a higher concentration of soluble molecules such as osteoprotegerin, C-Reactive Protein (CRP) and matrix metalloproteinases in comparison with subjects with the unique involvement of the skin [32]. Interestingly, higher levels of the pro-inflammatory chemokine C-X-C motif chemokine Ligand 10 (CXCL10), which is highly expressed in the diseased skin and targeted by IL-17 [33,34], can predict the evolution to PsA among patients affected by skin psoriasis [32].

### 2.3. Co-Morbidities and Complications of Psoriasis and Psoriatic Arthritis

Overall, both psoriatic and PsA patients show a similar spectrum of immune-mediated clinical manifestations and co-morbidities, which cooperate to reduce patients’ quality and duration of life [35,36]. Among them, cardiovascular diseases, diabetes and metabolic syndrome, Inflammatory Bowel Diseases (IBD) and osteoporosis, together with depression and fibromyalgia, play a major role. The prevalence of each of these manifestations may vary greatly between the two diseases: for instance, the risk of IBD occurrence and cardiovascular co-morbidities is increased in patients with PsA compared to Ps alone [36,37].

### 2.4. Histological Features of the Affected Skin and Synovium

Typical histologic features of the psoriatic skin include regular acanthosis, hypogranulosis, ectasia of the capillaries in the dermal papilla, and Munro microabscess; hypergranulosis may be also observed [38]. Skin involvement in PsA patients demonstrates the same characteristics but these patients also show different degrees of inflammatory changes within the synovial tissue of the affected joints. Synovial histopathology may be hugely heterogeneous but, generally speaking, it resembles the pattern observed in other spondyloarthropaties rather than the rheumatoid-associated synovitis [39]. A mild to moderate lining layer hyperplasia and tortuous blood vessels are predominant features, the latter being sustained by the expression of pro-angiogenic factors such as Vascular Endothelial Growth Factor (VEGF), and angiopoietins associated with a high expression of E-selectin [40]. As mentioned, the thickening of the lining layer secondary to macrophage hyperplasia and infiltration is usually less pronounced than in RA; however, pro-inflammatory cytokines released by macrophages (e.g., TNFα or IL-6) play a significant role in PsA too [39]. Compared to other inflammatory arthritis, the neutrophil component characterising the synovium in PsA is more abundant, mirroring the histological modifications observed in the psoriatic skin lesions [41].

In recent years, advances in the understanding of the synovial biology highlighted how the histological features of the diseased synovium during inflammatory joint diseases might be profoundly heterogeneous, defining alternative “pathotypes” that seem to associate, at least in RA, with clinical “phenotypes”. Synovial histological subsets can be categorised according to the pattern of infiltration of the immune cells. The presence of lymphocytic aggregates defines a so-called “lymphoid” pathotype, while the extensive presence of macrophages without clearly organised follicular structures depicts the “myeloid” subtype. The “pauci-immune” (or “fibroid”) synovial tissue is instead characterised by a scant infiltration of immune cells and a fibroblasts-rich stroma [42]. The same histological heterogeneity observed in RA synovium has also been detected in psoriatic synovial tissue [43]. An in-depth characterisation of the psoriatic synovium is currently ongoing in our group as part of the Pathobiology of Early Arthritis Cohort (PEAC, available online: http://www.peac-mrc.mds.qmul.ac.uk/) [44], a large multicentre observational study recruiting early arthritis, treatment-naïve patients affected by peripheral joint inflammation. The detailed assessment of the cellular and molecular infiltrate characterising the chronic inflammation of the synovial tissue in the course of PsA will be of critical importance for better comprehend the overlapping or divergent pathways driving the skin and/or the joint disease.

### 2.5. Pathogenic Immunologic Pathways Driving the Inflammation in Psoriasis and Psoriatic Arthritis

Several authors have recently reviewed the body of evidence evaluating the pathogenic pathways potentially responsible for the development and persistence of the clinical manifestations of Ps and PsA [7,45,46]. Within the skin, the inflammatory response usually follows the activation of CD8+ T cells by Antigen-Presenting Cells (APCs) such as Langerhans cells, which originate from the epidermis. The antigen(s) responsible for triggering the autoimmune reaction has not been identified yet. As per other autoimmune diseases, it has been suggested that the breakdown of the immune tolerance to potential antigens of infective origin (bacterial or viral) might constitute the initial stimulus, which subsequently leads to the chronic autoimmune process that characterises Ps [47,48]. A population of resident dermal γδ T cells has been shown to be able to proliferate in situ and to participate in the regulation of the skin immunity [49]. Despite numerous candidates have been proposed as autoantigens (e.g., type I keratin, papillomavirus 5, heat shock proteins), a clear proof of their pathogenic relevance in Ps is still missing. In addition to their role as initiators of the immune reaction, APCs can also produce and release pro-inflammatory cytokines such as IL-2 and IL-23, which drive the differentiation of naïve T lymphocytes into Th1 or Th17 cells. In turn, the massive infiltration of the psoriatic skin by activated T lymphocytes facilitates the secondary production of a broad range of inflammatory mediators able to induce the typical epidermal and dermal manifestations occurring in the course of Ps.

The central role of T lymphocytes in the pathogenesis of Ps has been acknowledged for years. While the importance of the Th1 subset has been historically recognised, a specific interest for the Th17 cells and its related IL-23/IL-17 axis has risen only in recent years; their critical function in Ps, however, is now well accepted and widely demonstrated [50].

In keeping with the relationship between Ps and the occurrence of PsA, it is plausible to hypothesise that the skin immune reaction might contribute and ultimately trigger the onset of the articular manifestations but whether PsA is driven by specific autoantigens shared between joint and skin is still under investigation. Histological evaluation of the psoriatic synovium showed the presence of both clonal and non-clonal T cells [51]. Interestingly, further studies revealed a relationship between skin and synovium T cells-clones [52], thus implying that a common antigen might drive the T cell response in both the target organs. Conversely, the expression of the Cutaneous Lymphocyte Antigen (CLA) in T cells present in the skin but not in the joints of the same patients with PsA [53], suggests a diverse antigen drive in the two compartments. With regards to the entheses, Dolcino et al. described cross-reactive PsA-specific antibodies directed against peptides expressed in both the psoriatic skin and the inflamed entheses [54]. Within the synovium, a major role is also played by the cytotoxic CD8+ cells, as suggested by their dominant presence in the synovial fluids of patients with PsA [55]. As mentioned, the histologic arrangement of the immune cells within the synovial tissue during PsA is considerably heterogeneous and includes, in some cases, the presence of Ectopic Lymphoid Structures (ELS). These lymphocytic structures, which can be found not only in autoimmune diseases but also in association with infections and cancer [56], have been shown to be able to self-sustain the local production of autoantibodies in the rheumatoid synovium [57]. Their specific features and functions within the psoriatic synovial membrane, however, have not been fully elucidated. Interestingly, clusters of DCs and T cells forming aggregates can also be found within the lesional psoriatic skin and have been shown to contribute to the chronicity of the disease [58]. More studies are now required to better understand their pathogenic role in skin psoriasis and their potential link with the development of PsA. In keeping with the key role played by the Th17 cells in driving the response within the psoriatic skin, numerous studies wondered about the expression and function of the Th17-related cytokines in the PsA-joints. With the advent of biologic agents targeting the IL-23/IL-17 axis, finding a definite answer to this question has become even more crucial to guide therapeutic decision making.

In the next section, we will review the pivotal role played by the IL-23/IL-17 axis in both Ps and PsA.

## 3. Pivotal Role of the IL-23/IL-17 Axis in Psoriasis and Psoriatic Arthritis

### 3.1. Key Cytokines and Receptors of the IL-23/IL-17 Axis

IL-17 is the eponymous cytokine produced by the Th17 subset of T lymphocytes. Other immune cells such as γδ T cells and Natural Killer (NK) cells can also synthesise IL-17. Th17 cells differentiate from naïve T cells in the presence of three potential combinations of cytokines: (i) IL-6 and Transforming Growth Factor-β (TGFβ), with the additional potentiating effect of IL-1β and TNFα; (ii) IL-21 and TGFβ; and (iii) IL-6, IL-1β and IL-23 [59]. The IL-17 family includes six members, namely IL-17A, B, C, D, E and F. Of them, IL-17A is the principal effector of the IL-17-related inflammatory activity in Ps and arthritis. A similarly significant role is also played by IL-17F, which is closely related to IL-17A and like this one can be secreted as heterodimer [60]. IL-17 can exert its effects by binding members of the IL-17-Receptors (IL-17R) family. Five different IL-17Rs exist, IL-17RA, RB, RC, RD and RE; the cellular responses to the IL-17A and IL-17F stimulation require both the constitutive expression of IL-17RA combined with the inducible expression of IL-17RC [61].

Th17 cells were first described as a subset of cells characterised by the common transcription factor ROR-γt and the signalling pathway Janus Kinase (JAK)-Signal Transducer and Activator of Transcription (STAT). Furthermore, Th17 cells share the same unique gene signature, which includes *IL17*, *IL6*, *TNF*, C-C motif Chemokine Ligand 20 (*CCL20*), Colony Stimulating Factor 2 (*CSF2*), *CCL22* and *IL23R*. In response to the IL-23 or IL-1β-mediated inflammation of the tissue, Th17 cells express and release signature effector cytokines such as IL-17, IL-21, IL-22 and Granulocyte Macrophage-Colony Stimulating Factor (GM-CSF). The Th17 family encompasses different cell types, all ubiquitously expressing ROR-γt and functioning via IL-23R, including subsets of Natural Killer T (NKT) cells and Innate Lymphoid Cells (ILCs). According to their function, Th17 cells can be categorised into host protective cells or pathogenic inflammatory cells. The ultimate role depends, on the one hand, by the cytokines promoting the Th17-differentiation and, on the other hand, by the relative balance of the effector molecules produced. Commonly, IL-23-activated Th17 cells trigger autoimmunity and chronic inflammation; conversely, TGFβ and IL-6 promote weakly pathogenic Th17 cells, important in tissue defence and integrity [62].

Among all T cell subsets, Th17 cells are the primary source of IL-22. This cytokine acts through a receptor composed of two subunits, namely IL-10R2 and IL-22R, and principally targets non-hematopoietic cells such as keratinocytes and epithelial cells. After binding the specific receptor, IL-22 exerts its effects through the activation of STAT3 and Mitogen-Activated Protein Kinase (MAPK). In homeostatic conditions, IL-22 plays a protective role and sustains the host defence favouring the regeneration of the epithelial barrier. On the contrary, during the course of inflammation, IL-22 is pathogenic and acts in synergy with other pro-inflammatory cytokines [63]. A recently described specific T lymphocytes population, named Th22, is characterised by the sole expression of IL-22 but not IL-17 and is strongly implicated in the epidermal immune system and inflammatory reactions [64].

IL-23 is constituted by two subunits, p19 and p40, which are linked by a disulphide bond. While the p19 subunit is an element unique to the IL-23, the p40 subunit is shared with IL-12. A significant amount of IL-23 is produced by keratinocytes and activated APCs, including Langerhans cells, macrophages and DCs [65]. When IL-23 binds to its receptor IL-23R, the complex recruits JAK2 and Tyk, members of the Janus family of tyrosine kinases, which in turn mediate the activation of the IL-23/IL23R and, eventually, the phosphorylation of the downstream STAT3. Once the IL-23 axis is activated, it induces the differentiation of the naïve T cells towards Th17. In particular, IL-23 is crucial not only for inducing the Th17 phenotype but also for enhancing within the Th17 subset the ability to become pathogenic [66,67]. Indeed, mice in which genes encoding key components of the IL-23/IL-17 pathway (*IL17*, *IL22*, *IL23A*) have been silenced are protected from both Ps and inflammatory arthritis, hence supporting the pathogenic role for the IL-23/IL-17 axis in these conditions [62]. Interestingly, these animals lack IL-17-producing T cells but show a regular Th1 component, thus suggesting that Th17 cells, but not Th1, are critical for the induction of the pathologic phenotype [68]. IL-23 has also been shown to promote the production of IL-17 by dermal γδ T cells, thus enhancing their pathogenic potential [69].

**Figure 1:**
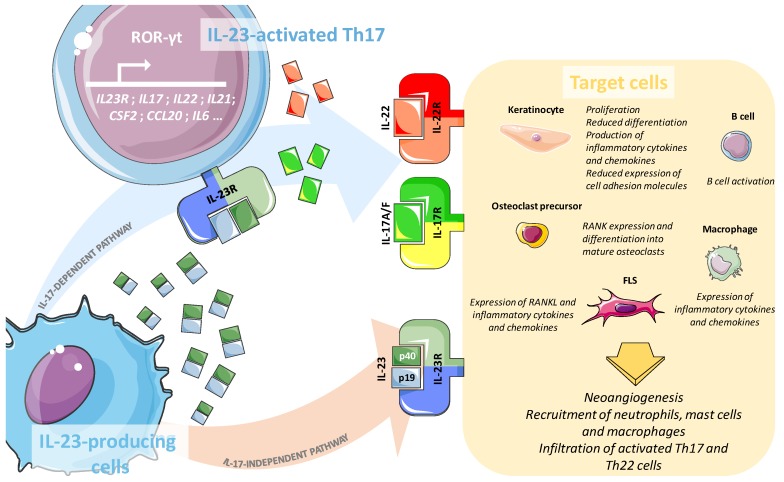
Main players of IL-23/IL-17 axis and their roles in the initiation and persistence of inflammation during Psoriasis and Psoriatic Arthritis. IL-23, mainly produced by dendritic cells, macrophages and keratinocytes acts on numerous target cells via either an IL-17-dependent or an IL-17-independent mechanism. In the first, IL-23 stimulates Th17 cells via IL-23R and induces the release of molecules such as IL-17 or IL-22. These, by binding their cognate receptors IL-17R or IL-22R, eventually activate the “effector cells” keratinocytes, B cells, osteoclast precursors, macrophages and FLS. Alternatively, the same subset of target cells can be directly challenged by the IL-23 in an IL-17-independent manner. The overall effect of the activation of the IL-23 pathway consists of the recruitment of inflammatory cells within the inflamed tissue. ROR-γt: Retinoic-acid-receptor-related Orphan Receptor-γt; Th: T helper; FLS: Fibroblasts Like Synoviocytes.

Upon activation, Th17 cells release effector molecules able to trigger a variety of target cells such as osteoclasts, B-cells and macrophages, which are responsible for the disease-specific inflammatory response [67,70]. IL-23 also plays a Th17-independent critical role in bone homeostasis and remodelling: by inducing the expression of the Receptor Activator of Nuclear factor κB (RANKL) in synovial fibroblasts and by up-regulating the expression of the RANKL-receptor RANK in osteoclast precursors, IL-23 eventually favours osteoclast differentiation and osteoclastogenesis [66]. The main players of the IL-23/IL-17 pathway are summarised and represented in Figure 1.

In human, GWAS and protein expression studies showed that IL-23p19 and p40, IL-22, IL-17, and their related receptors could be detected in psoriatic skin lesions and within the inflamed diseased synovium [63,71,72,73,74,75], hence suggesting that they might be the targets of treatments aiming at inhibiting the IL-23/IL-17 axis.

The complexity of the system, which acts through various mechanisms and involves numerous cells, offers multiple potential therapeutic sites for inhibition. To better define the mechanisms of action of these blocking agents and to try explaining the differential rates of response to this class of biologics in skin and joints, we will now focus on the specific pattern of expression and activity of the Th17 cytokines within the psoriatic skin and articular lesions.

### 3.2. Role of the IL-23/IL-17 Axis in the Psoriatic Skin

The pro-psoriatic activity of IL-23 has been suggested by the occurrence of psoriatic-like skin lesions in a mouse model of IL-23 administration intradermally. IL-23 can indeed act both independently of IL-17 by promoting the epidermal hyperplasia and activating the keratinocyte proliferation via the increase of Keratin 16 (K16) expression [76], and in association with IL-17 by enhancing dermal acanthosis, neutrophil recruitment and infiltration of IL-22 and IL-17-producing cells into the lesional skin [62]. Skin-resident cells such as keratinocytes, fibroblasts and endothelial cells, which express the receptor machinery for responding to IL-17 and IL-22 stimulation, react by up-regulating the expression of pro-inflammatory cytokines, chemokines (e.g., CXCL1 and CCL20), and anti-microbial peptides (such as LL-37 or β-defensins). Overall, IL-17 stimulation can increase keratinocytes proliferation, neo-angiogenesis, recruitment and activation of mast cells, neutrophils and macrophages, while reducing the expression of adhesion molecules thus favouring the disruption of the skin barrier. IL-22 also controls keratinocytes gene expression, enhancing the antimicrobial defence function, inhibiting their differentiation, and increasing their proliferation and mobility [63,77]. In animal models, IL-22 is required for the development of the Th17-mediated skin inflammation [78], and transgenic mice for IL-22 show a Ps-like skin phenotype [77]. Several studies have suggested that IL-22 can act in synergy with IL-17 or Interferon γ (IFNγ) through an IL-23-dependent mechanism [79] to amplify the inflammation observed in psoriatic skin [63].

### 3.3. Involvement of the IL-23/IL-17 Axis in Psoriatic Arthritis Synovium

Histologic and molecular studies have shown that IL-23 is expressed within the RA synovium and plays a pro-inflammatory role inducing both joint inflammation and bone destruction [73,80]. However, its expression seems to be restricted to high-inflamed synovial tissue rich in lymphoid aggregates [81]. Similarly, cytokines and receptors of the IL-17 family can be detected within the RA and PsA synovium but with profoundly different levels, thus suggesting that IL-17 expression could also be influenced by the synovial histologic features [75]. In keeping with these published data, we have observed an up-regulation of the IL-23/IL-17 cytokines and their receptors within lymphoid B-cells rich synovial pathotype in early untreated PsA patients part of the Pathobiology of Early Arthritis Cohort (PEAC) [44] (own unpublished data). Based on these observations, it is plausible to speculate that the synovial availability of the IL-23/IL-17 would influence the response to the biologic agents targeting this axis. Interestingly, a recent study demonstrated that the incomplete resolution of the inflammation in response to first-line anti-TNFα could trigger the emergence of the Th17 pathway [82].

Gene expression studies revealed that PsA synovium is more closely related to the psoriatic skin rather than the synovium of other inflammatory conditions like RA. However, notable differences in the level of expression of a few genes exist; for instance, the IL-17 signature is evidently stronger in the skin compared to the synovium [83]. In PsA, the synovial IL-23 expression is associated with higher indexes of disease severity [84], and the neo-vascularity of the tissue correlates with the recruitment of pathogenic IL-23/IL-17-producing CD4+ T cells into the joints [85]. However, whether the expression of the IL-23/IL-17 cytokines defines a specific clinical phenotype is not clear yet, and more studies in this field will clarify it.

Another important site of inflammation during PsA is represented by the connective tissue linking tendons and bone, called enthesis. Here, a population of resident Th17 cells, IL-17 and IL-22 producers, can influence the bone remodelling acting on osteoblasts in a STAT3 dependent manner, thus suggesting that the IL-23/IL-17 axis could be responsible for the typical bone formation observed in PsA entheses [86].

IL-22 is produced by synovial fibroblasts-like-cells (FLS) and macrophages [74] and is particularly raised in synovial fluids of PsA patients in comparison with osteoarthritis controls. In vitro, IL-22 can induce the proliferation of FLS in synergy with TNFα [87]. Even if a high number of IL-17/IL-22 producing CD4+ T cells is a common feature of both Ps and PsA patients, the relative frequency of this cell subset differs according to the anatomic site. Indeed, while a significantly high level of IL-22+ CD4+ cells characterises the psoriatic skin and can be detected in the circulation, vice-versa in synovial fluid and within the synovial tissue IL-22 expression is fairly scant [88].

Overall, these observations confirm the critical role played by the Th17 cells and their related cytokines IL-23/IL-17 in both Ps and PsA. However, while this pathway seems to be continuously expressed within the diseased skin, a rather “selective” presence characterises the joints, mostly dependent on the histological features of the tissue and the nature of the immune infiltrate. This different pattern of expression might be the reason for the divergent rates of clinical response to agents blocking the IL-23/IL-17 axis in skin and joints.

The Th17 pathway remains a valuable therapeutic target in both Ps and PsA, but, in the latter case, an in-depth assessment of the synovial tissue would be of critical importance to evaluate the availability of the target.

## 4. Towards a Personalized Use of the Biologics in Psoriatic Arthritis

### 4.1. The Divergent Response to Biologics Targeting the IL-23/IL-17 Axis in Psoriatic Skin and Joints

**Figure 2:**
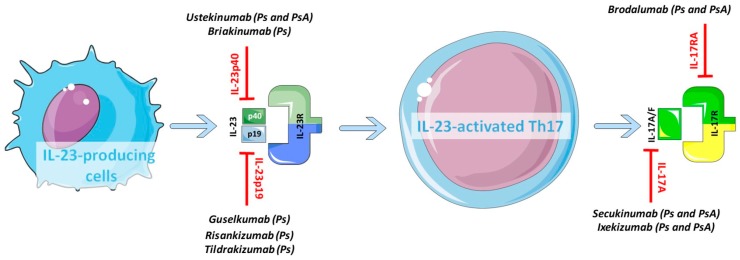
Biologics targeting the IL-23/IL-17 axis in Psoriasis (Ps) and Psoriatic Arthritis (PsA). Specific monoclonal antibodies targeting IL-23p40, IL-23p19, IL-17A or its receptor IL-17R have been developed and are currently used in clinical practice or still tested inclinical trials (see Table 1).

The introduction of the biologics agents into the therapeutic scenario carried a notable improvement of the clinical outcome of patients affected by skin psoriasis and inflammatory arthritis like PsA and RA. The availability of targeted treatment in association with the recognised importance of an early diagnosis [89] made the persistent low disease activity status and the disease remission achievable goals. In keeping with the well-established role played by Th17 cells in numerous autoimmune diseases including psoriatic disorders, a considerable effort has been made over the last years into the development of biologic drugs specifically designed to target this pathway, at various stages of its activation. Thus, a significant number of tailored molecules is currently available including ustekinumab, secukinumab, brodalumab, ixekizumab, guselkumab, risankizumab, briakinumab, and tildrakizumab (Figure 2). In Table 1, a list of the available biologics targeting the IL-23/IL-17 pathway has been provided, including both the already licensed and the molecules currently on trial. As discussed above, the IL-23/IL-17 axis is rather complex and offers multiple potential sites for action. The biologics have been categorised according to the specific molecule or sub-unit targeted: IL-12/23 p40, IL-23 p19, IL-17A and IL-17RA; for each agent, the results of the clinical trials evaluating its clinical efficacy have been summarised (Table 1). The minimal outcome improvement for regulatory approvals for skin response is usually assessed by the PASI75, which consists of a 75%-reduction of the PASI score from baseline to after treatment; for joint response, the minimal improvement considered clinically useful is 20% of the American College of Rheumatology (ACR) criteria.

Among the biologics targeting the p40 subunit, two molecules have been tested and approved: ustekinumab and briakinumab. Ustekinumab (CNTO 1275, Centocor Inc., Malvern, PA, USA) is a fully human monoclonal anti-IL12/23p40 antibody. Promising results from early small clinical studies [90,91] prompted two large randomised, double-blind, placebo-controlled trials, PHOENIX 1 (NCT00267969) [92] and PHOENIX 2 (NCT00307437) [93], aiming at defining efficacy and long-term safety profile. A total of 1996 patients with moderate-to-severe plaque psoriasis were randomised 1:1:1 into three groups to receive placebo or subcutaneous injections of ustekinumab (45 or 90 mg). The primary efficacy endpoint, consisting of PASI75, was reached in both trials, thus leading to the approval by the responsible authorising bodies. Post-hoc analyses of these clinical trials have subsequently confirmed the medium-to-long-term (3–5 years) efficacy and safety of the ustekinumab [94,95,96]. Notably, at five years, the proportions of patients obtaining a meaningful clinical outcome were satisfactory and consisted of 76.5% and 78.6% achieving PASI75 (treated with ustekinumab 45 and 90 mg, respectively), and still more than 50% reaching a PASI90 response [94]. Ustekinumab treatment was also beneficial in significantly improving nail psoriasis manifestations [97].

In keeping with the definite relationship between skin psoriasis and PsA, clinical trials assessing the effectiveness of ustekinumab in treating joint disease have been designed too. An initial multicentre, double-blind, placebo-controlled study (NCT00267956) carried out between North America and Europe recruited 146 patients with active PsA. Until the primary endpoint was assessed, the study was placebo-controlled. At 12 weeks, a significantly higher proportion of patients in the treated group achieved the ACR20 response (42% vs. 14%), but not the ACR50 and ACR70 [98].

The PSUMMIT1 study, phase III randomised placebo-controlled (NCT01009086), enrolled 615 patients with active PsA across Europe, North America and Asia-Pacific. Patients were randomly assigned to ustekinumab 45 mg or 90 mg, and placebo, and observed up to 52 weeks. Efficacy data were analogous to the study mentioned above. At week 24, 42.4% (dose 45 mg) to 49.5% (dose 90 mg) of patients reached the primary outcome (ACR20) compared to 22.8% responders in the placebo group. The efficacy of the ustekinumab on the skin was re-confirmed by significant improvement of the PASI score in the treated groups [99].

The results of the PSUMMIT 1 were also validated in the PSUMMIT 2 (NCT01077362), regarding both efficacy and safety [100]. Even if the joint response was significantly better in the ustekinumab group, once again the rate of patients achieving the minimal clinical response for approval (ACR20) did not exceed the 50%. Post-hoc analyses of PSUMMIT 1 and 2 also confirmed that ustekinumab was effective in improving the axial manifestations of PsA [101] and in preventing the radiologic progression [102].

Hence, ustekinumab has been recommended since 2015 not only for skin and nail psoriasis but also for peripheral psoriatic arthritis in Disease Modifying Anti-Rheumatic Drugs (DMARDs) non-responders patients [103].

Briakinumab (ABT-874, Abbott Laboratories, Chicago, IL, USA) is another fully human monoclonal antibody blocking the subunit p40. Since its efficacy was initially demonstrated in a phase II clinical trial (NCT00292396) [91], several phase III studies further proved effectiveness and safety. Gottlieb et al. [104] randomised 347 patients with mild-to-moderate plaque psoriasis to briakinumab, etanercept or placebo and demonstrated the superiority of the anti-IL12/23p40 over both the placebo and etanercept [primary outcome Physician Global Assessment (PGA) 0/1 and PASI75 response at week 12] (NCT00691964).

A phase III multicentre, randomized, double-blind clinical trial (NCT00679731) included 317 patients with active Ps assigned to receive either briakinumab or methotrexate. The proportion of patients achieving PASI75 and PGA 0/1 at week 24 and 52 was significantly higher in the briakinumab group compared to methotrexate, alongside with a greater number of serious adverse events [105]. Similar findings regarding not only the efficacy but also the safety were found in another 52-week randomised, double-blind, placebo-controlled trial (NCT00570986) [106]. Concerns on the safety profile of briakinumab prompted the analysis of the cumulative adverse events briakinumab-related recorded in five studies and an open-label extension publication. Increased rates of infections, malignancies and major adverse cardiac events were confirmed and, therefore, the application for the approval was withdrawn [107].

Guselkumab (CNTO 1959; TREMFYA; Janssen Biotech, Horsham, PA, USA) is a human monoclonal IgG1κ antibody binding the IL-23p19 subunit approved for the treatment of moderate-to-severe plaque psoriasis by the Food and Drugs Administration (FDA) [108]. The efficacy of guselkumab was firstly confirmed in a phase I first-in-human randomised, double-blind, placebo-controlled trial, which included 24 patients with moderate-to-severe plaque psoriasis. At 12 weeks, significantly more patients treated with subcutaneous guselkumab achieved the PASI75 response [109].

In VOYAGE1 (NCT02207231) and VOYAGE2 (NCT02207244), phase III multicentre randomised double-blind studies, guselkumab treatment was compared with placebo and adalimumab. In both the trials, >85% of the guselkumab-treated patients achieved PASI75 response (as compared with the PASI75 response to adalimumab ranging between 68.5% and 73.1%) [110,111].

Results from the NAVIGATE trial (NCT02203032) definitively confirmed previous findings and proposed guselkumab as an effective and well-tolerated therapeutic alternative for patients with moderate-to-severe psoriasis, including non-responders to a first line ustekinumab treatment [112].

Risankizumab (BI-655066, Boehringer Ingelheim, Ingelheim, Germany & Abbvie, North Chicago, IL, USA) is another high-affinity humanised antibody anti-IL23p19 [113]. Recent results of a 48-weeks multicentre randomised phase II trial (NCT02054481) showed a substantial superiority of the risankizumab over ustekinumab (blocking the p40 subunit). Better efficacy was demonstrated by the significantly higher proportion of patients achieving a 90% reduction in the PASI score at 12 weeks when treated with risankizumab (77%) compared to ustekinumab (40%), maintaining a similar safety profile [114]. Further studies are currently ongoing for confirming these promising data and for registration purposes (NCT02684370).

Tildrakizumab (Sun Pharmaceutical, Mumbai, India) is the third available molecule binding to and blocking the p19 subunit of the human IL-23. A randomised double-blind phase IIb trial (NCT01225731) including 335 patients with moderate-to severe-psoriasis confirmed the long-term efficacy (up to 52 weeks) of tildrakizumab (assessed by the PASI75 response) versus placebo [115]. Subsequent studies confirmed the superiority of the anti-IL-23 p19 blockade by tildrakizumab over both placebo (reSURFACE-1, NCT01722331) and Etanercept (reSURFACE-2, NCT01729754) [116].

Two molecules targeting IL-17 are currently in use: secukinumab and ixekizumab.

Secukinumab (Novartis Pharma AG, Basel, Switzerland) is a recombinant, high-affinity, fully human monoclonal anti-IL-17A antibody of the IgG1κ class. Its efficacy and safety were initially assessed in Ps, and later in PsA. Following encouraging results of preclinical and early clinical data, two large phase III double-blind 52-weeks trials (ERASURE and FIXTURE) were designed to evaluate secukinumab superiority to placebo. Both studies achieved the primary endpoint (PASI75 response) at 12 weeks. A greater number of adverse events was reported in the secukinumab group compared to placebo. However, the safety profile was comparable to etanercept [117,118].

The efficacy of secukinumab has been later investigated also in active PsA. The FUTURE 1 study (NCT01392326), a 2-years multicentre randomised double-blind placebo-controlled trial enrolled 606 patients with PsA, who were randomised to secukinumab or placebo. Both the primary endpoint (ACR20 response) and the secondary endpoints, which included the skin response PASI75 and PASI90, were met. Unfortunately, the rate of adverse events was particularly high [119,120]. Long-term efficacy and reduced radiologic progression were confirmed by following the FUTURE 1 patients up to 104 weeks [121,122]. The effectiveness of the IL-17 blockade in improving both joint and skin disease was further endorsed by the FUTURE 2 study (NCT01752634), a five-year randomised double-blind, multicentre placebo-controlled trial including 397 patients [123].

Thanks to these trials, secukinumab has been approved for the treatment of both moderate-to-severe plaque psoriasis and peripheral psoriatic arthritis in DMARDs-failure patients [124]. To note, in both FUTURE 1 and FUTURE 2, the percentage of patients fulfilling the ACR20 criteria of response did not exceed 53% at the highest dose.

Ixekizumab (LY2439821, TALTZ, Eli Lilly and Company, Indianapolis, IN, USA) is another recombinant humanised monoclonal antibody neutralising IL-17A. Three large multicentre, randomised, double-blind, placebo-controlled phase III trials assessed the efficacy and safety of ixekizumab [125].

In the study UNCOVER 1 (NCT01474512), involving 1296 patients, the superiority of ixekizumab over placebo on reducing PASI75 from baseline to week 12 was achieved. The UNCOVER 2 and 3 also confirmed the long-term efficacy of the molecule and the ability to induce high-level clinical responses (PASI90) [126]. Similarly to secukinumab, ixekizumab was also tested in PsA patients. SPIRIT-P1 (NCT01695239) was a three-year phase III randomised, double-blind, placebo and active-controlled clinical trial aiming at assessing the number of patients with active PsA achieving the ACR20 response at week 24 following treatment with ixekizumab in comparison with placebo and adalimumab. Ixekizumab was effective in reducing the activity and the radiologic progression of the joint disease, and 62.1% of patients achieved ACR20. In comparison, in the same study, the PASI75 skin response criteria were fulfilled by 79.7% of treated patients [127].

Brodalumab (AMG 827, Amgen, Thousand Oaks, CA, USA) is instead a human monoclonal IgG2 antibody developed to antagonise the IL-17RA.

In the AMAGINE 1 study (NCT01708590), a phase III double-blind randomised placebo-controlled study, 661 patients with moderate-to-severe psoriasis were recruited and randomized to receive brodalumab or placebo. The primary endpoint, namely the superiority of brodalumab over placebo measured by the percentage of patients achieving PASI75, was successfully achieved [128].

Two further phase III multicentre randomised double-blind studies named AMAGINE2 and AMAGINE3 (NCT01708603 and NCT01708629, respectively) compared the efficacy of brodalumab with placebo and ustekinumab. Not only the efficacy versus placebo was re-confirmed, but the highest dose of brodalumab was also more effective than ustekinumab in obtaining the PASI100 response at week 12 [129]. On the other hand, the use of the highest dose of brodalumab associated with more frequent and severe adverse events (possibly major depression and suicidal ideation).

Brodalumab has been approved for clinical use in the management of moderate-to-severe plaque psoriasis. Its potential use for treating PsA patients will be hopefully clarified by the results of a large phase III multicentre randomised double-blind placebo-controlled trial (NCT02024646) expected soon.

Overall, results from clinical trials showed that independently of the agent used, the amelioration of the skin psoriasis following the blockade of the IL-23/IL-17 axis was consistently more prominent than the joint response, with a larger percentage of patients achieving the PASI75, PASI90 and even PASI100 compared to proportion of patients fulfilling the ACR20, ACR50 or ACR70 criteria of response. On the other hand, the use of anti-TNFα agents gave more comparable rates of response between skin and joints. A plausible explanation for such organ-dependent divergence of response might be provided by comparative genomic profiling studies of paired synovium and skin lesions in PsA. Data in this field, indeed, demonstrated a stronger IL-17 gene signature into the diseased skin compared to the synovium, but a similar expression of the TNFα/IFNγ related genes [83]. In fact, different pathogenic mechanisms involving to a greater or lesser degree the IL-23/IL-17 pathway could ultimately sustain an alternative drive in skin and joint inflammation. Furthermore, the expression of the IL-23/IL-17-blockade targets within the psoriatic joints seems to be restricted to the synovial tissue showing active inflammation and ELS [75,81], therefore potentially identifying a “histologic” subset of patients more likely to respond to the inhibition of the Th17 pathway. Nowadays, in standard care, patients affected by skin psoriasis or PsA not adequately responding to standard systemic treatment and conventional synthetic DMARDs, are eligible to start one of the available biologic agents among both the anti-TNF and the anti-IL-23/IL-17 agents. According to the UK NICE (National Institute for health and Care Excellence) guidelines, psoriatic patients without joint involvement can receive either anti-TNF or anti-IL-23/anti-IL-17 drugs, depending on the age of the patient and the severity of the skin disease. In PsA patients, on the other hand, TNF-blocking agents represent first line biologic therapy, reserving antagonists of the IL-23/IL-17 axis as the second line. The sequential use of these molecules is based on a trial-and-error approach and does not take into account any of the histologic features of the synovial tissue. A more rational choice of the biologic agent with the highest chances to be efficacious may be guided by the histologic characteristics of the diseased synovium and by the availability of the therapeutic target. As already hypothesised in the context of RA [130], such a stratified approach in PsA would ultimately increase the rate of responders, limit the exposure to side effects and reduce the economic burden related to the use of the biologic less likely to be effective in some patients.

### 4.2. Novel Perspectives and Future Drugs

To further potentiate the clinical efficacy of the IL-23/IL-17 blockade, several new strategies are currently under investigation in pre-clinical and early phase clinical trials.

#### 4.2.1. Targeting ROR-γt

ROR-γt is the key transcription factor that regulates the differentiation of the Th17 cells. Its pharmacological blockade can inhibit the Th17 maturation and modulate the expression of the Th17 gene signature, thus representing an attractive novel therapeutic strategy. Small molecules targeting ROR-γt have shown interesting effects in in vivo models of Ps-like inflammation induced by the Toll-Like-Receptor (TLR) 7/8 agonist Imiquimod [133]. Future investigations and clinical trials will help to understand the potential benefits and safety of the ROR-γt-blockade in patients with Ps and PsA.

#### 4.2.2. Targeting JAK/STAT

The JAK/STAT system is involved at different levels of the IL-23/IL-17 signalling pathway; for example, the receptors of both IL-22 and IL-23 rely on JAK to initiate the downstream signalling into the cells and utilise mainly STAT3 to govern the Th17 effector responses. Recently, Welsch et al. extensively reviewed the existing literature about the small molecules designed to target this pathway [134]. In pre-clinical studies involving synovial tissue from PsA patients, tofacitinib, a JAK1/3 inhibitor, has been shown to be able to down-regulate the secretion of pro-inflammatory cytokines, thus limiting the inflammation within the synovium [135].

Phase I to III clinical trials are investigating the role of JAK inhibitors in the treatment of Ps and PsA; tofacitinib, for instance, has been proved to be significantly more effective than placebo in PsA patients non-responders to conventional synthetic DMARDs [136].

#### 4.2.3. Targeting Multiple Cytokines and Novel Anti-Inflammatory Pathways

A simultaneous targeting of both the Th17-associated and non-associated inflammatory cytokines could represent another possible winning strategy to improve the treatment of skin and joint inflammation in Ps and PsA. For instance, bispecific monoclonal antibodies recognising IL-17A and IL-1β have been recently tested by Li and colleagues and showed to be effective to ameliorate the outcome of animal models of arthritis [137] while human studies are underway.

Finally, over the last few years, novel critical antagonists of the IL-1 family, namely IL-37 and IL-38, have been discovered. These cytokines mediate immunosuppressive effects in in vivo models of skin [138] and joint inflammation [139,140] by potentially reducing the expression of the Th17-associated cytokines [141] and attenuate the inflammation triggered by IL-1, IL-18, IL-36 and TLR-agonists [142,143]. Overall, exploiting the IL-37/IL-38 pathway could represent an innovative therapeutic strategy for controlling inflammation in Ps and PsA.

## 5. Conclusions

The IL-23/IL-17 axis plays an undoubtedly critical role in the development of both the cutaneous and the articular clinical manifestations associated to psoriasis. Nevertheless, tissue-specific differences in the activation and expression of this pathway have been highlighted by genetic and histologic studies comparing psoriatic skin and synovium. Consistently, the diversified rates of response to the IL-23/IL-17 blockade observed in skin and joints further sustain a clinical significance of this divergence in the expression of the Th17-associated cytokines.

The number of the biologic agents targeting the IL-23/IL-17 is continuously growing, and plenty of new molecules antagonising alternative pathways are currently under investigations in pre-clinical studies too. Conceivably, the armamentarium for treating Ps and PsA will soon include several new weapons and, therefore, the research of valuable predictors of response to alternative therapeutic strategies will be of critical importance.

## Figures and Tables

**Table 1:** Randomized clinical trials on inhibitors of the IL-23/IL-17 axis in Psoriasis and Psoriatic Arthritis. Ust: Ustekinumab; Bri: Briakinumab; MTX: Methotrexate; Gus: Guselkumab; Ada: Adalimumab; Ris: Risankizumab; Til: Tildrakizumab; Eta: Etanercept; Sec: Secukinumab; Ixe: Ixekizumab; Bro: Brodalumab; PASI: Psoriasis Area and Severity Index; ACR: American College of Rheumatology; IGA: Investigator’s Global Assessment.

Agents	Name of the Study and Reference	Condition	Trial Phase	Treatment Arm	Control Arm	Primary Endpoint	
						Skin PASI75 at Week 12 Unless Otherwise Specified	Joint-Related ACR20 Unless Otherwise Specified
*Anti-IL12/23p40*							
**Ustekinumab (CNTO 1275, Stelara)**	**PHOENIX 1** *NCT00267969* [92]	Psoriasis	III	Ust 45 mg (*n* = 255) Ust 90 mg (*n* = 256)	Placebo (*n* = 255)	Ust 45 mg 67.1% Ust 90 mg 66.4% Placebo 3.1%	-
	**PHOENIX 2** *NCT00307437* [93,95]	Psoriasis	III	Ust 45 mg (*n* = 409) Ust 90 mg (*n* = 411)	Placebo (*n* = 410)	Ust 45 mg 66.7% Ust 90 mg 75.7% Placebo 3.7%	-
	**PSUMMIT1** *NCT01009086* [99]	PsA	III	Ust 45 mg (*n* = 205) Ust 90 mg (*n* = 204)	Placebo (*n* = 206)	Ust 45 mg 57.2% Ust 90 mg 62.4% Placebo 11.0%	At week 24: Ust 45 mg 42.4% Ust 90 mg 49.5% Placebo 22.8%
	**PSUMMIT 2** *NCT01077362201* [100]	PsA	III	Ust 45 mg (*n* = 103) Ust 90 mg (*n* = 105)	Placebo (*n* = 104)	At week 24: Ust 45 mg 51.3% Ust 90 mg 55.6% Placebo 5%	At week 24: Ust 45 mg 43.7% Ust 90 mg 43.8% Placebo 20.2%
**Briakinumab (ABT-874, Ozespa)**	*NCT00691964* [104]	Psoriasis	III	Bri 200 mg × 2 then 100 mg (*n* = 138)	Etanercept 50 mg twice-weekly (*n* = 141) Placebo (*n* = 68)	Bri 81.9% Eta 56.0% Placebo 7.4%	-
	*NCT00679731* [105]	Psoriasis	III	Bri 200 mg × 2 then 100 mg (*n* = 154)	MTX 5 to 25 mg weekly (*n* = 163)	(At week 24) Bri 81.8% MTX 39.9%	-
	*NCT00710580* [131]	Psoriasis	III	Bri 200 mg × 2 then 100 mg (*n* = 138)	Etanercept 50 mg twice-weekly (*n* = 141) Placebo (*n* = 68)	Bri 80.6% Eta 39.6% Placebo 6.9%	-
	*NCT00570986* [106]	Psoriasis	III	Bri 200 mg × 2 then 100 mg (*n* = 981)	Placebo (484)	Bri 80.7% Placebo 4.5%	-
*Anti-IL23p19*							
**Guselkumab (CNTO 1959; Tremfya)**	**NAVIGATE** *NCT02203032* [112]	Psoriasis	III	Ust non-responder patients at Week 12 (total *n* = 268): Gus 100 mg (*n* = 135) Ust (*n* = 133)	Ust responder patients at Week 12 (*n* = 585)	Visits at which patients achieved IGA 0/1 and >2-grade improvement: Gus 1.5 ± 1.6 Ust 0.7 ± 1.3	-
	**VOYAGE-1** *NCT02207231* [110]	Psoriasis	III	Gus 100 mg (*n* = 329)	Placebo (weeks 0, 4, 12) then Gus 100 mg (weeks 16, 20) (*n* = 174) Ada 80 mg (*n* = 334)	PASI75 at week 16: Gus 91.2% Placebo 5.7% Ada 73.1%	-
	**VOYAGE-2** *NCT02207244* [111]	Psoriasis	III	Gus 100 mg (*n* = 496)	Placebo (weeks 0, 4, 12) then Gus 100 mg (weeks 16, 20) (*n* = 248) Ada 80 mg (*n* = 248)	PASI75 at week 16: Gus 86.3% Placebo 8.1% Ada 68.5%	-
**Risankizumab (BI 655066)**	*NCT02054481* [114]	Psoriasis	II	Ris 18 mg (*n* = 43) Ris 90 mg (*n* = 41) Ris 180 mg (*n* = 42)	Ust (*n* = 40)	PASI90 at week 12: Ris 90 and 180 mg 77% Ust 40%	-
	**UltIMMa-1** *NCT02684370* (Not yet completed)	Psoriasis	III	Ris	Ust Placebo	Not yet completed	-
**Tildrakizumab (MK-3222)**	**reSURFACE-1** *NCT01722331* [116]	Psoriasis	III	Til 100 mg (*n* = 309) Til 200 mg (*n* = 308)	Placebo (*n* = 155)	Til 100 mg 62% Til 200 mg 64% Placebo 6%	-
	**reSURFACE-2** *NCT01729754* [116]	Psoriasis	III	Til 100 mg (*n* = 307) Til 200 mg (*n* = 314)	Placebo (*n* = 156) Eta (*n* = 313)	Til 100 mg 61% Til 200 mg 66% Placebo 6% Eta 48%	-
*Anti-IL17A*							
**Secukinumab (AIN457, Cosentyx)**	**ERASURE** *NCT01365455* [118]	Psoriasis	III	Sec 150 mg (*n* = 245) Sec 300 mg (*n* = 245)	Placebo (*n* = 248)	Sec 150 mg 71.6% Sec 300 mg 81.6% Placebo 4.5%	-
	**FIXTURE** *NCT01358578* [118]	Psoriasis	III	Sec 150 mg (*n* = 327) Sec 300 mg (*n* = 327)	Placebo (*n* = 326)	Sec 150 mg 67.0% Sec 300 mg 77.1% Placebo 4.9%	-
	**FUTURE1** *NCT01392326* [119]	PsA	III	Sec 75 mg (*n* = 202) Sec 150 mg (*n* = 202)	Placebo (*n* = 202)	PASI75 at week 24: Sec 75 mg 64.8% Sec 150 mg 61.1% Placebo 8.3%	ACR20 at week 24: Sec 75 mg 50% Sec 150 mg 50.5% Placebo 17.3%
	**FUTURE2** *NCT01752634* [123]	PsA	III	Sec 75 mg (*n* = 99) Sec 150 mg (*n* = 100) Sec 300 mg (*n* = 100)	Placebo (*n* = 98)	PASI75 at week 24: Sec 150 mg 48% Sec 300 mg 63% Placebo 16%	ACR20 at week 24: Sec 75 mg 29% Sec 150 mg 51% Sec 300 mg 54% Placebo 15%
**Ixekizumab (LY2439821, *Taltz*)**	**UNCOVER-1** *NCT01474512* [126]	Psoriasis	III	Ixe 80 mg q2wk (*n* = 433) Ixe 80 mg q4wk (*n* = 432)	Placebo (*n* = 431)	Ixe 80 mg q2wk 89.1% Ixe 80 mg q4wk 82.6% Placebo 3.9%	-
	**UNCOVER-2** *NCT01597245* [126]	Psoriasis	III	Ixe 80 mg q2wk (*n* = 351) Ixe 80 mg q4wk (*n* = 347)	Eta 50 mg twice-weekly (*n* = 358) Placebo (*n* = 168)	Ixe q2wk 89.7% Ixe q4wk 77.5% Eta 41.6% Placebo 2.4%	-
	**UNCOVER-3** *NCT01646177* [126]	Psoriasis	III	Ixe 80 mg q2wk (*n* = 385) Ixe 80 mg q4wk (*n* = 386)	Eta 50 mg twice-weekly (*n* = 382) Placebo (*n* = 193)	Ixe q2wk 87.3% Ixe q4wk 84.2% Eta 53.4% Placebo 7.3%	-
	**SPIRIT-P1** *NCT01695239* [127]	PsA	III	Ixe 80 mg q2wk (*n* = 103) Ixe 80 mg q4wk (*n* = 107)	Ada 40 mg q2wk (*n* = 101) Placebo (*n* = 106)	PASI75 at week 24: Ixe q2wk 79.7% Ixe q4wk 71.2% Ada 54.4% Placebo 10.4%	ACR20 at week 24: Ixe q2wk 62.1% Ixe q4wk 57.9% Ada 57.4% Placebo 30.2%
*Anti-IL17RA*							
**Brodalumab (AMG 827, Siliq)**	**AMAGINE-2** *NCT01708603* [129]	Psoriasis	III	Bro 140 mg (*n* = 610) Bro 210 mg (*n* = 612)	Ust 45 mg (*n* = 300) Placebo (*n* = 309)	Bro 140 mg 67% Bro 210 mg 86% Ust 70% Placebo 8%	-
	**AMAGINE-3** *NCT01708629* [132]	Psoriasis	III	Bro 140 mg (*n* = 629) Bro 210 mg (*n* = 624)	Ustekinumab 45 mg (*n* = 313) Placebo (*n* = 315)	Bro 140 mg 69% Bro 210 mg 85% Ust 69% Placebo 6%	-
	*NCT01516957*	PsA	II	Bro 280 mg (*n* = 56) Bro 140 mg (*n* = 57)	Placebo (*n* = 55)	-	Bro 280 mg 39% Bro 140 mg 37% Placebo 18%
	**AMVISION-2** *NCT02024646* (Not yet completed)	PsA	III	Bro 140 mg Bro 210 mg	Placebo	Not yet completed	Not yet completed

## Abbreviations

Ps	Psoriasis
PsA	Psoriatic Arthritis
CASPAR	ClASsification criteria for Psoriatic Arthritis
RA	Rheumatoid Arthritis
Th	T helper
ROR-γt	Retinoic-acid-receptor-related Orphan Receptor-γt
HLA	Human Leukocyte Antigen
NF-κB	Nuclear Factor-Kappa B
TNF	Tumor Necrosis Factor
IFIH1	Interferon-Induced with Helicase C Domain 1
GWAS	Genome-Wide Association Studies
MTX	Methotrexate
PASI	Psoriasis Area and Severity Index
CRP	C-Reactive Protein
CXCL10	C-X-C motif chemokine Ligand 10
IBD	Inflammatory Bowel Disease
VEGF	Vascular Endothelial Growth Factor
PEAC	Pathobiology of Early Arthritis Cohort
APC	Antigen-Presenting Cells
CLA	Cutaneous Lymphocyte Antigen
ELS	Ectopic Lymphoid Structure
NK	Natural Killer
TGFβ	Transforming Growth Factor-β
IL-17R	IL-17 Receptors
JAK	Janus Kinase
STAT	Signal Transducer and Activator of Transcription
CCL20	C-C motif chemokine Ligand 20
CSF2	Colony Stimulating Factor 2
GM-CSF	Granulocyte Macrophage-Colony Stimulating Factor
NKT	Natural Killer T
ILC	Innate Lymphoid Cells
MAPK	Mitogen-Activated Protein Kinase
DC	Dendritic Cells
RANKL	Receptor Activator of Nuclear factor κB
K16	Keratin 16
IFNγ	Interferon γ
ACR	American College of Rheumatology
PGA	Physician Global Assessment
FDA	Food and Drugs Administration
DMARDs	Disease Modifying Anti-Rheumatic Drugs
NICE	National Institute for health and Care Excellence
Ust	Ustekinumab
Bri	Briakinumab
Gus	Guselkumab
Ada	Adalimumab
Ris	Risankizumab
Til	Tildrakizumab
Eta	Etanercept
Sec	Secukinumab
Ixe	Ixekizumab
Bro	Brodalumab
IGA	Investigator’s Global Assessment
